# New option: targeting RNase J and RNase HI in the fight against multi-drug-resistant tuberculosis

**DOI:** 10.1097/MS9.0000000000001859

**Published:** 2024-03-04

**Authors:** Priyatam Khadka, Januka Thapaliya

**Affiliations:** a Tribhuvan University, Trichandra Multiple Campus, Kirtipur, Nepal; b State University of New York, Upstate Medical University, Syracuse, NY

Tuberculosis remains a recalcitrant global health issue despite being discovered a century ago and powerful antibiotics being developed. 10.6 million cases of active tuberculosis were reported to the WHO in 2022; of those cases, 1.3 million succumbed to death making tuberculosis a leading cause of fatality^1^. Its’ increasing transmissibility, rising resistance to two main antibiotics (isoniazid inhibits cell wall formation, and rifampicin inhibits transcription), and tendency to persist in latent forms are major reasons for this catastrophe^2^. In 2022, 410 000 new cases of multi-drug-resistant tuberculosis (MDR-TB) including extensively drug-resistant (XDR) were recorded; hence, it is critical to enhance TB therapies and halt the spread of resistance^1^. Typically, there are four primary challenges which underlie the shortcomings of TB treatment: drug tolerance fuels and synergises with drug resistance; sequential drug and regimen development is intrinsically slow, despite the development of tools to rationally prioritise regimens early in the cascade; a surprisingly small number of drugs have been tested as preventive therapy of latent tuberculosis; and TB treatment takes a lot longer to cure than other bacterial infections^3^. Therefore, drug development priorities should be focused on elucidating biological knowledge gaps, prioritising combination therapy, breaking the tolerance-resistance link and restricting possible reactivation.

GyrA/B, ATP synthase, QcrB, FadD32, Pks13 and MmpL3 are high-dimensional biological readouts that have attracted a lot of scrutiny in drug discovery^4^. Unfortunately, a few of the drug candidates that inhibited some of the preceding targets had toxicity or insufficient in-vivo activity. A reliable crutch for combating MDR bacteria involves targeting fundamental biochemical pathways such as transcription and translation. By using these cellular pathways, bacteria can regulate the expression of specific genes, which allows them to quickly adapt to changes in their growing environment^5^. Therefore, it would be possible to create new targets if we could control and leverage the significant differences in mRNA degradation complexes between species^6^. Ribonucleases (RNases), RNA helicases, and glycolytic enzymes form an extremely organised protein complex called RNA degradosome, that degrades RNA^7^. The roles of RNA degredosomes including specific RNases’ may vary depending on the bacteria type, either Gram-positive or Gram-negative. Usually, the RNase E gene is present in gram-negative bacteria, while RNase J and RNase Y are present in most gram-positive bacteria; these ribonucleases are essential in global mRNA decay^8–10^. *Mycobacterium tuberculosis* (MTB), unlike these organisms, contains both RNase J and RNase E^11^. Polynucleotide phosphorylase (PNPase) and ATP-dependent RNA helicase (RhlE) are also involved in MTB degradosome pathways, as discovered recently.

## RNase J

About half of all bacterial species contain the prokaryotic ribonuclease RNase J, which is essential for both RNA processing and degradation^12^. RNase has been implicated, in recent literature, as the mechanism governing the growth and survival of bacteria^13,14^. Two different paralogs, J1 and J2, have been identified so far. RNase J differs from all other ribonucleases in that it can function as a processive 5’ exonuclease as well as an endonuclease^15^.

RNase J, a crucial component of the MTB degradation complex, significantly impacts growth, survival, and drug tolerance^13,14,16^. Antibiotic resistance in MTB was previously linked to mutations in RNase J, a bacterial enzyme involved in the processing and degradation of RNA. Bao *et al.* ^17^ observed growth rate, colony morphologies, aggregation and motility of *Mycobacterium smegmatis* were dramatically slowed down by RNase J knockdown, and this was reversed when the RNase J gene was supplemented. The loss of RNase J can lead to drug tolerance, which is beneficial for managing drug stress during tuberculosis therapy but may also lead to high-level drug resistance in clinical settings^18^. Changes in gene expression that take place in the absence of RNase J are the cause of the increased survival. The significance of this work lies in the description of a novel mechanism that bacteria can employ to evade antibiotic treatment. Not limited to MTB, in several pathogenic bacterial species, including *B. subtills, C. diphtheriae, S. aureus, and H. pylori,* which naturally lack RNase E, RNase J takes its role in the mRNA degradation process. It has been shown that Rnase J disproportionately mutated in drug-resistant MTB therefore possibilities of further mutations should not be undermined. Focusing on RNase J could be a novel therapeutic strategy for treating MTB infection; however, validation with several studies is crucial.

## RNase HI

For a bacterium to retain its genomic stability, RNase HI is an essential enzyme. RNase HI can resolve the supercoiling or uncoupling of DNA during translation, and hybrids of DNA and RNA can arise during transcription^19,20^. This may lead to the formation of R-loops, endangering the stability of bacteria’s genomes. RNase HI activity corrects this cellular dysfunction, primarily through the selective hydrolysis of the RNA strand in the RNA/DNA hybrid^21,22^.

Mycobacteria have a bi-functional RNase HI enzyme called RnhC, which includes an N-terminal domain with RNase HI activity and a C-terminal domain with cobalamin-P phosphatase activity^23–25^. Specifically in mycobacteria, the synthesis of vitamin B12 depends on RNase HI activity; the simultaneous deletion of two RNase HI genes, rnhC and rnhA, is fatal, suggesting the essentiality of RNase HI activity^23,25,26^. Al-Zubaidi *et al.* ^22^ in their experiment revealed the deletion of RnhC leads to R-loop accumulation and genome topological stress, enhancing anti-tubercular drug killing. These results provide credence to the idea that RNase HI is a potential therapeutic target and imply that strains developing resistivity to rifampicin could be restricted^22,25^. Not limited to Mycobacteria, the therapeutic implications of Rnase HI activity were observed to be promising in HIV and *Escherichia coli (*rifampicin-resistant and streptomycin-resistant strains)^27,28^. High-affinity RNase HI inhibitors reduce MIC_50_ of potent anti-tubercular drugs(rifampicin, streptomycin, isoniazid, moxifloxacin) due to synergistic effects with first-line antibiotics—eventually reducing off-target effects and reverse resistance^22^. Al-Zubaidi *et al.* ^22^ demonstrated ten RNase HI inhibitors to inhibit MTB at a concentration of less than 100 μM, of which NSC353720 was a potent inhibitor while NSC99726 was the weakest.

In the days ahead of research, the potential for Rnase J and Rnase Hi mutations as well as the potential for inhibitor resistance should not be undermined. On a positive note, our evaluation recommends RNase J and Rnase HI as promising druggable targets for mycobacteria; consideration of several prospects is required, however.

## Ethical approval

Ethics approval was not required for this editorial.

## Consent

Informed contest was not required for this editorial.

## Source of funding

Not applicable.

## Author contribution

P.K. designed the manuscript, reviewed the literature, and prepared the article for submission. J.T. helped with literature reviews, gave a concept of the research paper, and critically reviewed the manuscript. Both authors read and approved the final manuscript.

## Conflicts of interest disclosure

The authors declare that they have no competing interests.

## Research registration unique identifying number (UIN)

Not applicable.

## Guarantor

Priyatam Khadka.

## Data availability statement

Not applicable.

## Provenance and peer review

Not commissioned, externally peer-reviewed.
